# Beamed UV sonoluminescence by aspherical air bubble collapse near liquid-metal microparticles

**DOI:** 10.1038/s41598-020-58185-2

**Published:** 2020-01-30

**Authors:** Bradley Boyd, Sergey A. Suslov, Sid Becker, Andrew D. Greentree, Ivan S. Maksymov

**Affiliations:** 1 Department of Mechanical Engineering, University of Canterbury, Christchurch, 8041 New Zealand; 2 Department of Mechanical Engineering, Texas A&M University, College Station, TX USA; 3 Department of Mathematics, Swinburne University of Technology, Hawthorn, Victoria 3122 Australia; 4 Australian Research Council Centre of Excellence for Nanoscale BioPhotonics, School of Science, RMIT University, Melbourne, Victoria 3001 Australia; 5 Centre for Micro-Photonics, Swinburne University of Technology, Hawthorn, Victoria 3122 Australia

**Keywords:** Biophysics, Nanophotonics and plasmonics

## Abstract

Irradiation with UV-C band ultraviolet light is one of the most commonly used ways of disinfecting water contaminated by pathogens such as bacteria and viruses. Sonoluminescence, the emission of light from acoustically-induced collapse of air bubbles in water, is an efficient means of generating UV-C light. However, because a spherical bubble collapsing in the bulk of water creates isotropic radiation, the generated UV-C light fluence is insufficient for disinfection. Here we show, based on detailed theoretical modelling and rigorous simulations, that it should be possible to create a UV light beam from aspherical air bubble collapse near a gallium-based liquid-metal microparticle. The beam is perpendicular to the metal surface and is caused by the interaction of sonoluminescence light with UV plasmon modes of the metal. We estimate that such beams can generate fluences exceeding 10 mJ/cm^2^, which is sufficient to irreversibly inactivate most common pathogens in water with the turbidity of more than 5 Nephelometric Turbidity Units.

## Introduction

The ability of UV-C light (200–280 nm) to inactivate bacteria, viruses and protozoa is widely used as an environmentally-friendly, chemical-free and highly effective means of disinfecting and safeguarding water against pathogens responsible for cholera, polio, typhoid, hepatitis and other bacterial, viral and parasitic diseases^1^. UV-C light inactivates pathogens through absorption of radiation energy by their cellular RNA and DNA prompting the formation of new bonds between adjacent nucleotides. This results in a photochemical damage that renders pathogens incapable of reproducing and infecting^1^.

However, pathogens can recover from photochemical damage when the initial UV dosage (fluence) is not sufficiently high^1^. For example, the fluence must be 5 and 10 mJ/cm^2^, respectively, to inactivate 99% and 99.9% of *Giardia* and *Cryptosporidium* pathogens^2^. These specifications are for water purified from solid particles larger than 5–10 *μ*m [turbidity of 5 Nephelometric Turbidity Units (NTU)]^2^. Otherwise, particles can shield pathogens from the UV light, thereby allowing many pathogens to recover and infect.

The filtration of natural water presents significant challenges for remote communities and developing nations^2^. Moreover, filtered water, dissolved iron, organic salts and the pathogen population itself absorb UV-C light. Therefore, a 50% UV radiation loss has been accepted as suitable for practical use^1^.

To enable UV disinfection of turbid water, we suggest to use the effect of sonoluminescence—the emission of broadband UV light in acoustically-induced collapse of air bubbles in water^3–5^. Air bubbles suitable for sonoluminescence are often present in natural water^6,7^ and their concentration can be increased, for example, by using bubble diffusers^8^. We show that such bubbles could act as compact sources of germicidal radiation located several optical wavelengths away from pathogens. This means that shielding of pathogens by particles suspended in water would be greatly reduced, but small distances travelled by UV-C light between the source and pathogens would result in negligible absorption losses.

**Figure 1:**
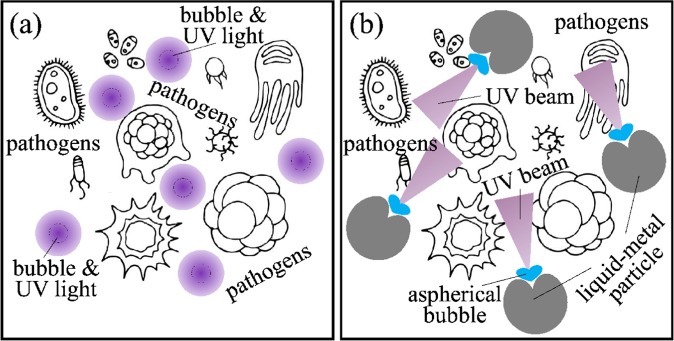
(**a**) The collapse of a single spherical air bubble in water creates isotropic, low-fluence radiation unsuitable for UV germicidal irradiation, (**b**) the collapse of an aspherical bubble near a liquid-metal particle results in the deformation of the liquid-metal surface and interaction of the emitted light with UV plasmon modes of the microparticle. This creates directed, high-fluence UV-C light beams that should be capable of inactivating 99.9% of most common pathogens.

However, conventional sonoluminescence of spherical air bubbles produces isotropic radiation^3,4^, which means that the fluence of light generated by any single collapse event is low compared to that required for UV germicidal irradiation [Fig. 1(a)]. We show that it should be possible to create a directed UV-C light beam via sonoluminescence of air bubbles collapsing near microparticles made of non-toxic and environmentally-friendly gallium-based alloys^9^, which could easily be removed from disinfected water and, if required, could also be used to purify water contaminated with heavy metals^10^. The resulting beam would be perpendicular to the metal surface because of the resonant interaction of the emitted sonoluminescence light with UV-C plasmons in the microparticle^11^ [Fig. 1(b)]. We show that such beams should generate UV-C fluences exceeding the thresholds required to irreversibly inactivate 99.9% of most common pathogens. Because the melting point of gallium-alloy metals is lower than room temperature^9,12^, we also show that the germicidal effect could be achieved with both liquid and solid metal microparticles.

## Results

### Spherical bubble collapse in unbounded water

The collapse of a spherical bubble in unbounded liquid is described by the bubble radius *R*(*t*) that oscillates around the equilibrium radius *R*_0_. We employ a Rayleigh-Plesset model that is commonly used in the context of sonoluminescence^4^ and can also be extended to analyse bubble collapse near a rigid boundary^13^

1 $$\begin{array}{ccc}\rho \left(R\ddot{R}+\frac{3}{2}{\dot{R}}^{2}\right) & = & \left[{p}_{g}-{p}_{0}-P\left(t\right)\right]-\frac{4\eta \dot{R}}{R}-\frac{2\sigma }{R}+\frac{R}{v}\frac{d{p}_{g}}{dt},\\ {p}_{g}\left(t\right) & = & \left({p}_{0}+\frac{2\sigma }{{R}_{0}}\right)\frac{{({R}_{0}^{3}-{a}^{3})}^{\gamma }}{{\left[R{\left(t\right)}^{3}-{a}^{3}\right]}^{\gamma }},\end{array}$$

where $\dot{R}$ and $\ddot{R}$ are the first and second time derivatives of *R*(*t*), *P*(*t*) = −*p*_max_sin(2*πft*) with the frequency *f* and time *t*, and *p*_0_ = 101.3 kPa. We solve this equation by using a fourth order Runge-Kutta scheme^14^. The material parameters are^4,14^: density *ρ* = 1000 kg/m^3^, sound velocity *v* = 1500 m/s, viscosity *η* = 1.002 × 10^−3^ Pa s, surface tension *σ* = 0.0728 N/m, *γ* = 1.4 and *a* = *R*_0_∕8.5. The temperature inside the bubble is given by the following formula with *T*_0_ = 298 K^4^

2 $$T\left(t\right)={T}_{0}\frac{{({R}_{0}^{3}-{a}^{3})}^{\left(\gamma -1\right)}}{{\left[R{\left(t\right)}^{3}-{a}^{3}\right]}^{\left(\gamma -1\right)}}.$$

The vapour pressure of water (*p*_*v*_ ≈ 3.17 kPa) is neglected in our model because it is approximately 30 times smaller than the ambient pressure *p*_0_. Therefore, omitting this term in the Rayleigh-Plesset equation does not change the results of our analysis qualitatively. We also refer the interested reader to a relevant discussion in Appendix C of ref. ^15^.

Sonoluminescence can be observed in a limited range of bubble parameters that enable gas exchange processes, high temperatures and bubble shape stability^4^. Atmospheric air contains ~1% of argon and high-temperature sonochemical reactions inside the collapsing bubble lead to a rapid removal of oxygen and nitrogen thereby leaving only the noble gas inside the bubble and enabling the emission of light^4^. According to the Blake threshold criterion for sonoluminescence^4^, *R*_0_ has to be greater than ${R}_{0}^{C}=C\sigma /({p}_{max}-{p}_{0})$. With *C* ≈ 0.77 and *p*_max_ = 1.7*p*_0_^4^, we obtain ${R}_{0}^{C}\approx 0.8$ *μ*m. Hence, in the following we assume that *R*_0_ = 1 *μ*m. We also ensure that *f* < *f*_0_, where *f*_0_ is the main resonance frequency of the air bubble in water (*f*_0_*R*_0_ ≈ 3.26 m/s).

**Figure 2:**
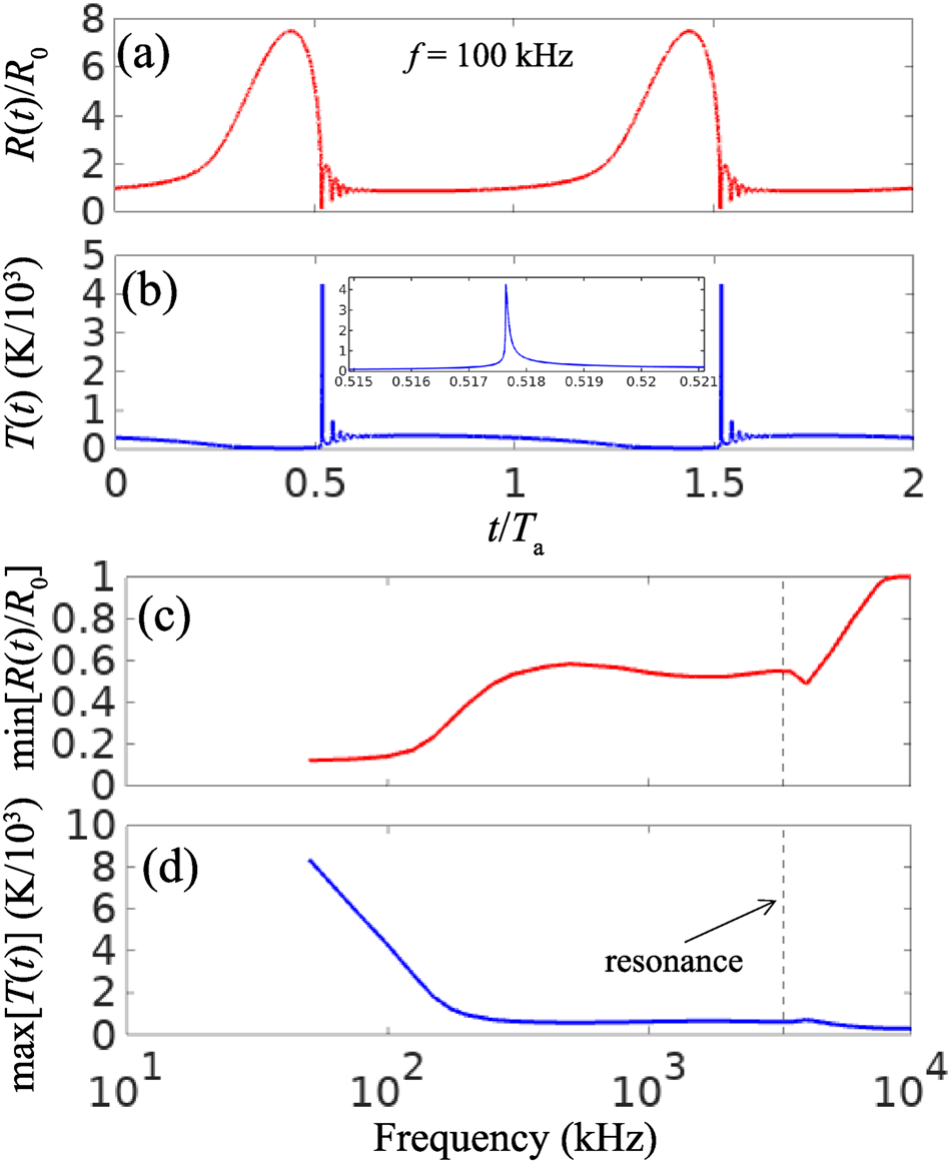
(**a**) Calculated non-dimensional bubble radius *R*(*t*)/*R*_0_ (*R*_0_ = 1 *μ*m) and (**b**) temperature *T*(*t*) of the bubble as a function of time *t* given in units of the acoustic wave period *T*_*a*_. The acoustic wave frequency is *f* = 100 kHz and the peak pressure amplitude is *p*_max_ = 1.7*p*_0_. The inset shows the zoom of the main temperature peak. (**c**) Minimum bubble radius min[*R*(*t*)∕*R*_0_] and (**d**) peak temperature max[*T*(*t*)] of the bubble as functions of *f*. The broken vertical lines show the main bubble resonance frequency *f*_0_.

As a representative example, Fig. 2(a) shows the dynamics of the non-dimensional bubble radius *R*(*t*)/*R*_0_ for *f* = 100 kHz. We observe a large excursion of *R*(*t*) from *R*_0_ followed by a steep collapse with a series of sharp afterbounces. The temperature of the bubble dramatically increases when *R*(*t*)/*R*_0_ reaches its minimum [Fig. 2(b)], which results in the sonoluminescence pulse. The duration of this pulse is defined by the full width at half maximum (FWHM) of the temperature peak^3^. The minimum radius min[*R*(*t*)/*R*_0_], the peak temperature max[*T*(*t*)] of the bubble and the FWHM of the light pulse depend on the acoustic frequency *f* [Fig. 2(c,d)]. The value of max[*T*(*t*)] varies slowly when *f* > *f*_0_, remains approximately constant for *f* ≈ *f*_0_ and increases quickly at *f* < *f*_0_ when the collapse becomes more violent.

### Aspherical bubble collapse near metal microparticles

When the bubble oscillates near a solid surface, it flattens near the surface^15–18^. At the collapse stage, a water jet develops through the centre of the bubble toward the surface^15–18^. The collapse near a solid surface also results in stable sonoluminescence^3,19^.

The latter property is important for the application envisioned in this work because the stability of sonoluminescence implies that pathogens would be continuously exposed to germicidal UV radiation. In the course of multiple expansion-collapse cycles some bubbles stop producing UV light, but others, which were not previously involved in the process, start contributing to sonoluminescence. Thus, on average the UV radiation fluence delivered to pathogens should be stable over a complete disinfection cycle.

We analyse an aspherical bubble collapse near spherical gallium-alloy particles of 50–100 *μ*m radius, which can be fabricated by means of a self-breakup of a liquid metal jet^9^. We assume that an initially spherical bubble with *R*_0_ = 1 *μ*m nearly touches the particle—the distance between the bubble centre and the metal surface is 1.1 *μ*m. Because *R*_0_ is 50–100 times smaller than the radius of the particle, we simplify our model by considering a planar 100 *μ*m-thick liquid-metal layer.

We use a high-order numerical method for solving fully-compressible multiphase inviscid flow equations^15,16,20^

3 $$\begin{array}{ccc}{\partial }_{t}({\alpha }_{i}{\rho }_{i})+\nabla \cdot ({\alpha }_{i}{\rho }_{i}{\boldsymbol{u}}) & = & 0,\\ {\partial }_{t}(\rho {\boldsymbol{u}})+\nabla \cdot (\rho {\boldsymbol{u}}\otimes {\boldsymbol{u}}+p{\boldsymbol{I}}) & = & 0,\\ {\partial }_{t}E+\nabla \cdot [{\boldsymbol{u}}(E+p)] & = & 0,\\ {\partial }_{t}{\alpha }_{1}+{\boldsymbol{u}}\cdot \nabla {\alpha }_{1} & = & 0,\\ {\partial }_{t}({\alpha }_{1}+{\alpha }_{2})+{\boldsymbol{u}}\cdot \nabla ({\alpha }_{1}+{\alpha }_{2}) & = & 0,\end{array}$$

where *i* = 1, 2, 3, *α*_*i*_ are the volume fractions of bubble gas, water, and liquid metal (∑_*i*_*α*_*i*_ = 1), *ρ* = ∑_*i*_*α*_*i*_*ρ*_*i*_ is the density, ***u*** is the velocity vector, *p* is the pressure, *E* = ∑_*i*_*α*_*i*_*E*_*i*_ is the total energy, and ***I*** is the identity matrix.

These equations combined with an equation of state (EOS) for the mixture of bubble gas, water, and liquid metal define the compressible-multiphase system^20^. We neglect viscous forces because they are significantly smaller than the pressure forces driving the collapse. The influence of surface tension is also neglected because the primary force driving the collapse is due to the difference between the pressure inside the bubble and the acoustic pressure^20^. The density of the liquid metal is 6360 kg/m^3^ and the parameters for the EOS are taken from^21^. The presence of the 1–3 nm-thick gallium oxide layer^11^ is neglected because it does not cause qualitative changes in fluid-mechanical and optical properties of the metal.

**Figure 3:**
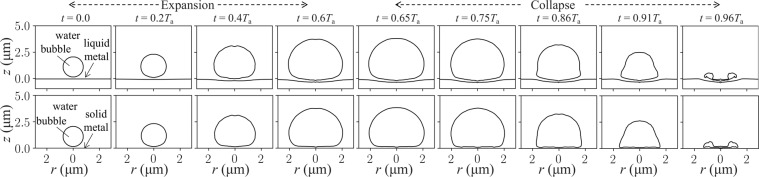
Representative axisymmetric profiles of an air bubble during its expansion and collapse near the liquid metal (top row) and solid metal (bottom row). *T*_a_ is the period of the sinusoidal acoustic wave (*f* = 1.5 MHz, *p*_max_ = 2*p*_0_^16^) incident along the *z*-axis toward the metal surface. A concave shape assumed by the liquid-metal surface as a result of the collapse will allow us to focus sonoluminescence light into a more intense beam compared to that near a flat solid-metal surface.

 Figure 3 shows the profiles of the bubble and metal surface during one period *T*_a_ of the *f* = 1.5 MHz sinusoidal acoustic wave that triggers the expansion-collapse cycles of the bubble and the deformation of the liquid-metal surface. A toroidal bubble is formed in the end of the collapse stage, which is also the instance when the sonoluminescence light is emitted^3,19^. This behaviour closely resembles that of the bubble located near the solid metal surface, but the solid metal is not deformed.

The formation of a toroidal bubble is accompanied by a water micro-jet impinging the metal surface^15–18^. We calculate the pressure developed by the micro-jet near the *solid metal* to be  ~7 MPa, which agrees with experimental data^17,18^.

When modelling the motion of the *liquid-metal* surface, we assume that due to a periodicity of the bubble expansion-collapse process it would regain its initial shape in the beginning of each cycle. We predict the jet pressure at the liquid metal surface to be ~26 MPa. This value may be somewhat overestimated because of a numerical instability arising in the end of the collapse stage, but experimental data suitable for validation of this prediction are currently not available.

It can be shown that the development of a water jet would not cause significant permanent damage to the liquid-metal surface. By analogy with the solid-metal surface, which is warranted because of the similarity between the respective bubble dynamics in Fig. 3 and the pressures developed by the water jets, noticeable damage to the liquid-metal surface would mostly be observed after several hours of continuous cavitational destruction^7,22–25^. It is also known that prolonged irradiation of macroscopic liquid gallium alloy particles with MPa-level pressure ultrasound may break them into smaller particles^11,26^. However, for this to occur, irradiation times should be of order of several minutes^11,26^, which is much longer than the existence time of the water jet that, in turn, constitutes a small fraction of the period of oscillation of an acoustic wave driving the collapse. Moreover, smaller liquid-metal particles are known to quickly coalesce forming macroscopic particles unless their surface is modified or special measures are taken to remove the surface oxide layer^9,26^. However, such special conditions are not present in our scenario.

The numerical instabilities grow as the frequency *f* decreases because the collapse becomes more violent. Thus, we follow the theoretical prediction^27^ of consistent aspherical collapse shapes at *f* < *f*_0_ ≈ 3.26 MHz. Similar to the radius of spherical bubbles *R*(*t*) (Fig. 2), the volume of aspherical bubbles *V*(*t*) exhibits larger excursions from *V*_0_ followed by steeper collapses when *f* is decreased^27^. This enables us to use the frequency dependence of the temperature of spherical bubbles [Fig. 2(d)] to predict the temperature of aspherical ones.

### Model of sonoluminescence

To simulate the emission of the sonoluminescence light, we use a three-dimensional finite-difference time-domain (FDTD) method based on Maxwell’s equations^14^. The fluid-mechanical and FDTD models have two very different time scales associated with a low acoustic frequency *f* and high UV light frequency. Therefore, the optical solution reaches a quasi-steady state before the shapes of the bubble and liquid metal have changed substantially. Hence, in the FDTD model we can use the instantaneous snapshots of the bubble and liquid-metal profiles from Figs. 2 and 3.

We use the blackbody emission model^4^ to simulate the creation of sonoluminescence light by a collapsing bubble^4^. The UV-C light fluence predicted by this model is about two orders of magnitude larger than experimental values for the same parameters^4^. Nevertheless, it remains suitable for analysis of experimental results^28^, but its simplicity and intuitiveness enable us to demonstrate the germicidal effect of the UV-C light.

A blackbody of temperature *T* produces a spectral radiance

4 $${L}_{\lambda }\left[T\right]=\frac{2h{c}^{2}}{{\lambda }^{5}}\frac{1}{{\rm{e}}{\rm{x}}{\rm{p}}\left(\frac{hc}{\lambda {k}_{{\rm{B}}}T}\right)-1}$$

with the Planck and Boltzmann constants *h* ≈ 6.63 × 10^−34^ m^2^kg/s and *k*_B_ ≈ 1.38 × 10^−23^ m^2^kg/(s^2^K), and the speed of light in vacuum *c* ≈ 3 × 10^8^ m/s. The spectral radiant power is calculated by integrating *L*_*λ*_ over the projected bubble surface and all solid angles^4^. For a spherical bubble it is *Φ*_*λ*_(*t*) = 4*π*^2^*R*(*t*)^2^*L*_*λ*_[*T*(*t*)] with *R*(*t*) and *T*(*t*) drawn from Fig. 2.

The FDTD method can readily simulate wide-spectrum signals such as *L*_*λ*_. However, it does not allow for a quantitative control of the emitted power, which means that output energy quantities are expressed in arbitrary units. To calculate the fluence in real physical units, we exploit the linearity of Maxwell’s equations and first simulate the spatial pattern of the emitted light. This pattern corresponds to the profile of radiant emittance, a radiometric term that is equivalent to intensity in optics, expressed in arbitrary units. Then, we semi-analytically calculate the total amount of the radiant power as $\Phi (t)={\int }_{{\lambda }_{1}}^{{\lambda }_{2}}{\Phi }_{\lambda }(t)\ {\rm{d}}\lambda$ with *λ*_1_ = 200 nm and *λ*_2_ = 280 nm. The time integration of Φ(*t*) gives the optical energy in Joules, but the spatial pattern of the emitted light allows us to define the area through which the radiation passes. This allows us to calculate the fluence as the energy delivered per unit area.

The spatial resolution of the FDTD mesh is 5 nm. In the UV-C band, the liquid and solid state gallium alloys share the same complex dielectric permittivity described by a Drude model^11^, which means that liquid and solid metal surfaces with identical profiles would equally affect UV-C light. The refractive index of water is 1.366^29^.

**Figure 4:**
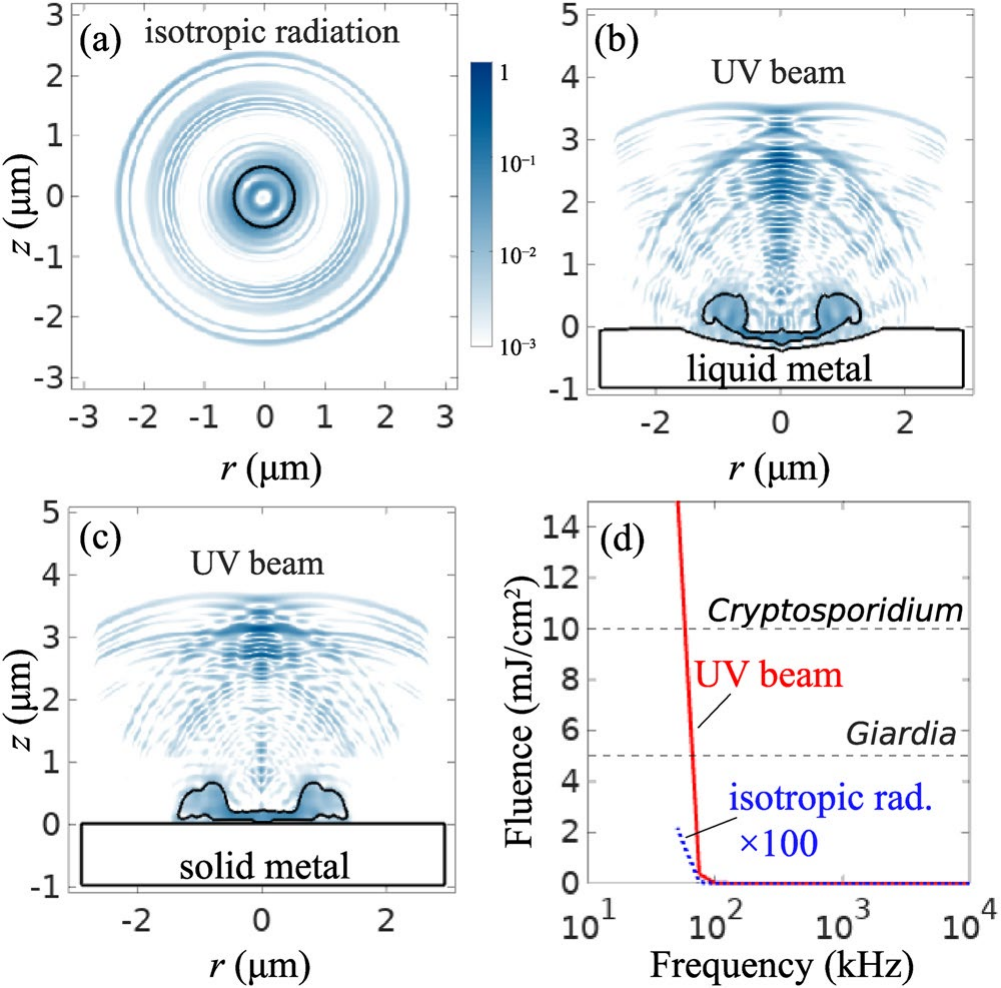
Spatial patterns of light emitted by (**a**) spherical bubble in unbounded water, (**b**) aspherical bubble near the *liquid-metal* surface, and (**c**) aspherical bubble near the *solid-metal* surface. All patterns are normalised to the maximum magnitude in (**b**). (**d**) UV radiation fluence as a function of the acoustic frequency *f*. The fluence of isotropic radiation is multiplied by 100. The fluence required for the inactivation of *Giardia* and *Cryptosporidium* pathogens is indicated.

 Figure 4 shows the steady-state spatial pattern of sonoluminescence light at *f* = 1.5 MHz. The radius of the spherical bubble is 500 nm [Fig. 2(c)] and the aspherical profiles are taken from Fig. 3. By virtue of the Fourier transform, this time-domain wave packet carries the spectral radiance *L*_*λ*_ for *λ* = 200–280 nm. A spherical bubble produces isotropic radiation while its aspherical counterpart produces a beam near both liquid and solid metal surfaces. The beam arising near a deformed liquid-metal surface acting as a concave mirror is more intense compared to that near a flat solid surface.

The liquid-metal surface has excellent optical properties in a broad spectral range covering the UV, visible and near-infrared wavelengths^11,30–33^. Significantly, in the UV range, both liquid and solid-state gallium alloy structures support strong plasmon resonances leading to enhanced focusing of light produced by sources located near the metal surface^11,30– 32,34^. This property is used in the current work.

The energetics of sonoluminescence produced by spherical and aspherical bubbles are similar^3,19^, which in the framework of the blackbody model requires *T*(*t*) to follow the same trend. Hence, we use *T*(*t*) from Fig. 2 to calculate the UV light fluence generated by both spherical and aspherical bubbles. For the spherical bubble, we use the area of an imaginary sphere with the radius *R*_0_ = 1 *μ*m. For the aspherical bubble, we take the radius (~75 nm) and the cross-sectional area of the beam from the spatial profile in Fig. 4(b).

The above approximation serves the purpose of this work demonstrating the feasibility of UV sonoluminescence beams. Indeed, we consider a bubble that initially nearly touches the liquid-metal surface and then develops and maintains a quasi-hemispherical shape over a significant portion of its expansion-collapse cycle (Fig. 3). It is well-known that quasi-hemispherical bubbles collapsing near a solid boundary emit UV light and develop a water jet in the late stage of the collapse^3,19^. This behaviour was noticed in early experiments on cavitation and was extensively used in the literature to model the dynamics of cavitation near solid boundaries^35–38^. This further justifies our assumption of similar energetics of sonoluminescence produced by spherical and aspherical bubbles.

 Figure 4(d)shows that the fluence generated by both spherical and aspherical bubbles dramatically increases at *f* < 100 kHz. However, only the formation of a directed beam near the metal microparticles results in the irreversible inactivation of pathogens. The improvement of the germicidal effectiveness is due to the sharp increase in temperature *T* as *f* decreases [Fig. 2(d)]. The wavelength of the peak of the blackbody radiation scales as *λ*_*p*_ = *b*/*T* with *b* ≈ 2.9 × 10^−3^ K m, which implies that *λ*_*p*_ shifts to the UV-C band when *f* is decreased.

As shown in Fig. 4(d), the UV radiation fluence delivered to pathogens can be increased simply by decreasing the acoustic frequency *f*. This has two important implications. Firstly, from the point of view of potential applications, fluences above 40 mJ/cm^2^ are required to inactivate various viruses and destroy bacterial spores^1,2^. Hence, by varying the acoustic frequency one should be able to control the fluence level targeting specific pathogens present in water. Secondly, even though the current use of a simple blackbody model in our analysis may result in the overestimation of the fluence delivered to pathogens at a given ultrasound frequency (this can be checked by employing a more accurate volume emitter model^4^), the increase of fluence due to lowering the frequency is expected to be sufficient to compensate for such a model overestimation so that the realistic output of 40 mJ/cm^2^ is maintained. We also note that in practice pathogens would be exposed to higher fluences produced by an ensemble of bubble. Therefore, the maximum fluence delivered to pathogens could be increased by increasing the concentration of bubbles in water.

## Discussion

We have suggested that the collapse of air bubbles and sonoluminescence near liquid-metal particles should result in the generation of UV light beams capable of inactivating pathogens contaminating drinking water. In contrast to conventional UV light water disinfection systems^1^, a setup based on our approach would be suitable for disinfecting turbid water. This is essential for applications in developing countries where the filtration of natural water presents significant challenges.

For example, conventional UV light disinfection systems are inefficient when water is not properly purified from microscopic solid particles: such particles can shield pathogens from the UV light thereby allowing many of them to recover and infect^1^. A similar shortcoming is inherent to the solar UV light disinfection method^2^, which also requires a prolonged exposure of water to sun light ranging from 6 hours to several days depending on weather conditions. The recovery of pathogens is less likely to occur in the approach suggested in this work, where air bubbles collapsing near liquid-metal particles dispersed in the bulk of water act as compact sources of germicidal radiation located only several optical wavelengths away from pathogens, which greatly reduces the shielding of pathogens by turbidity particles suspended in water.

In addition to a shielding effect, conventional UV light disinfection methods suffer from another shortcoming. Iron and organic salts dissolved in water as well as the pathogen population itself absorb up to 50% of UV light in conventional UV water disinfection systems^1^. This doubles energy consumption in UV light generation. The cost of that is normally acceptable in developed countries but could be a limiting factor for economically developing nations. The water disinfection system proposed here should be more affordable for remote communities and developing countries, also benefiting developed nations, because equipment required for a its practical realisation would include simple, reliable and inexpensive devices such as a generator of microbubbles, ultrasound transducers and power supplies. This equipment could also be combined with water treatment systems using gas bubble injection^8^ and ultrasounic radiation^39^.

We calculate that the UV beams produced by the aspherical air bubble collapse can generate fluences exceeding 10 mJ/cm^2^, which is sufficient to irreversibly inactivate most common pathogens in water with the turbidity of more than 5 Nephelometric Turbidity Units. We expect that a water disinfection system based on our approach would be suitable for the treatment of small batches and/or low flows of water at the local community level.

Liquid-metal particles remaining in disinfected water can be removed and reused in further disinfection cycles by using mechanical, chemical or electrochemical methods^40–42^. One could also convert them into porous filters for further improvement of water quality^10^.

Metal alloys based on the eutectic mixture of gallium and indium^9^ and also tin (e.g. Galinstan^12^) could be used as the constituent material of the liquid-metal particles. These materials are approved by the Food and Drug Administration and similar organisations, they are non-toxic and environmentally-friendly and are used, for example, in mercury-free analog clinical thermometers that are inexpensive and reliable^43^. The actual cost of the liquid metal is much lower and it could be decreased by using a mass-production technique such as self-breakup of a liquid metal jet^9^.

The plasmonic properties of gallium alloys can also be used to resonantly enhance the intensity of light by about two orders of magnitude^11,30–33^. This concerns not only the sonoluminescence light, but also light emitted by external sources such as lasers. In particular, lasers are used to generate bubbles suitable for experiments on sonoluminescence^44–46^ and it has also been shown that the generation of such bubbles can be controlled by using plasmonic particles^47^. Furthermore, the resonant plasmonic enhancement of the intensity of light opens up an opportunity to compensate for a decrease in the intensity of sonoluminescence light caused by the effect of non-sphericity of the collapse of laser-induced bubbles^44–46^.

Finally, the proposed water disinfection scheme should also be more energy- and cost efficient than water boiling^48^ or water chlorination^49^ and ozonation^50^. For example, a reliable disinfection of water is possible after a minimum of 20 minutes of continuous boiling. Moreover, before the boiled water can be used it needs sufficient time to cool during which it can be recontaminated since in a tropical environment bacteria proliferate at a fast rate. Handling of boiling water can also lead to serious injuries such as burns.

The use of chlorine and ozone also poses a significant challenge for remote communities and developing nations. For example, all forms of chlorine are highly corrosive and toxic. Therefore, storage, shipping, and handling of chlorine pose a significant risk and require special safety arrangements^49^. Disinfection systems using ozone are also expensive and complex^50^. In contrast, a system based on our approach should be safe, inexpensive as well as easy to install and operate.

## Methods

Standard fourth-order Runge-Kutta scheme^14^ and finite-difference time-domain (FDTD) methods^11,51^ were used, respectively, to solve the Rayleigh-Plesset equation and Maxwell’s equations with appropriate initial and boundary conditions. In the FDTD simulations, the standard Drude model was employed to fit experimental values of the dielectric permittivity of the liquid metal^11^.

The dynamics of the acoustically-driven bubble was modelled by using a high-order, fully-compressible, multiphase flow model^15,16,20^. An immersed, moving, reflective boundary was used to simulate the oscillations of the active face of an ultrasound transducer resulting in the acoustic field^15,16,20^. The model presented in ref. ^15^ was modified to capture the bubble growth prior to the bubble collapse and extended to represent a three-fluid system including air, water, and liquid metal^20^.

## Data Availability

The datasets generated during and/or analysed during the current study are available from the corresponding author on reasonable request.
